# Reabilitação Baseada em Exercícios para Pacientes Pré e Pós Transplante de Órgãos Sólidos

**DOI:** 10.36660/abc.20220373

**Published:** 2022-07-29

**Authors:** Rosane Maria Nery

**Affiliations:** 1 Hospital de Clínicas de Porto Alegre Grupo de pesquisa em cardiologia do exercício (CardioEx) Porto Alegre RS Brasil Grupo de pesquisa em cardiologia do exercício (CardioEx), Hospital de Clínicas de Porto Alegre, Porto Alegre, RS – Brasil

**Keywords:** Transplantes de Orgãos/reabilitação, Imunologia de Transplantes, Atividade Física, Transplante de Rim, Transplante de Fígado

Nas últimas décadas, inúmeros avanços na área do transplante de órgãos sólidos (TOS) culminaram em uma maior sobrevida dos pacientes, repercutindo em um aumento considerável no número de transplantes realizados no mundo.^1,2^ O TOS é uma intervenção que salva vidas em portadores de doença cardíaca, pulmonar, renal ou hepática. Apesar de os receptores obterem uma melhora, tanto na capacidade funcional quanto na qualidade de vida (QV), estas ainda não equivalem aos mesmos níveis dos indivíduos saudáveis.^3^

O longo período de espera, causado pela falta de doadores de órgãos, muitas vezes faz com que os pacientes não estejam preparados para o transplante, não apenas fisicamente, mas também mentalmente. Além dos fatores de risco cardiovasculares clássicos, ainda temos a falta de adesão do paciente aos programas, déficits de conhecimento sobre as regras de conduta após transplante, não aceitação do novo órgão, medo da rejeição, falta de uma rotina de exercícios físicos, estratégias de enfrentamento e questões de saúde ocupacional e direito social.^4,5^

A reabilitação é uma parte essencial do cuidado contemporâneo de pacientes antes e depois do transplante. O que se busca é a melhora da sobrevida do enxerto e a redução de mortes por infecção/rejeição. Os programas de reabilitação têm objetivos profiláticos e terapêuticos, atendendo as recomendações de manter melhorias na QV, reduzir a morbidade por doenças cardiovasculares e melhorar a sobrevida a longo prazo em receptores de transplante.^6^ Por isso, uma maior atenção deve ser dada às intervenções pós-cirúrgicas que auxiliam no manejo individualizado destes pacientes e que podem resultar em um melhor prognóstico.^7^

Entre as intervenções não farmacológicas pós-cirúrgicas, o treinamento físico merece destaque, estando associado à melhora significativa da tolerância ao exercício e a capacidade funcional, redução da incapacidade, e diminuição da morbidade cardiovascular e mortalidade. Este tem se mostrado benéfico também em vários grupos de doenças crônicas que podem levar a TOS. Sabe-se que existe uma limitação da capacidade de realização de exercícios físicos em indivíduos pré TOS, sendo que, a maioria dos estudos tem se concentrado em candidatos a transplante de coração e pulmão.^8^ Entretanto, pessoas com doença renal ou hepática crônicas também demonstram limitações na capacidade de exercício pré-transplante, muitas vezes devido a consequências secundárias do desuso, como fraqueza muscular, e não como consequência de seu processo primário de doença.^9^ Nestes indivíduos a limitação do consumo de oxigênio de pico parece estar relacionada à disfunção muscular periférica e não a fatores centrais, como limitações cardiovasculares ou respiratórias.^10^

Apesar das evidências mostrando os potenciais benefícios do exercício físico para pacientes tanto pré quanto pós TOS, existe uma grande carência de locais que ofereçam este atendimento de forma mais global. Isso piorou muito após o evento do COVID-19 que restringiu mais ainda o acesso aos centros de reabilitação. Uma grande proporção de receptores de TOS praticam baixos níveis de exercício físico e enfrentam barreiras para serem fisicamente ativos.

O estudo de Ribeiro et al.,^11^ sugere uma estratégia onde, após avaliação pré-participação, na ausência de contraindicação cardiovascular e de acordo com a preferência do paciente, ele pode optar por realizar seu programa de exercícios na academia do hospital, na academia comunitária ou em casa. Esse modelo permite que mais pessoas consigam se engajar num programa de exercícios, recebendo orientação de um profissional habilitado, tendo consultas periódicas presenciais ou por tele consulta.^12^ Os resultados encontrados pelos autores reforça a importância do programa supervisionado, mas enfatiza que qualquer tipo de tratamento será eficaz, desde que o paciente se proponha executá-lo.^11^ Além das limitações descritas pelos autores, o fato de terem abordado, num mesmo estudo, receptores de órgão diferentes, pode ter influenciado os resultados dos programas de reabilitação baseados em exercícios (PRBE), uma vez que conforme doença crônica prévia, os receptores apresentam heranças fisiopatológicas que podem influenciar diretamente nos resultados da reabilitação.

Ainda são necessários estudos maiores e bem controlados de exercício físico, que incluam especificamente candidatos a transplante, que possam propor orientações específicas sobre a dose de exercício e duração do programa para atingir os melhores benefícios.^13^ Estudos futuros neste tópico devem se concentrar, também, nos efeitos do exercício durante o período na lista de espera, preparando o paciente para o evento do transplante e desfechos clínicos pós-transplante precoce.

Para a população mundial, o acesso aos centros de reabilitação ainda é muito pequeno, principalmente em locais com baixo nível de estrutura e recursos na saúde, baixo nível socioeconômico e cultural da população. Nosso desafio é saber como usar os conhecimentos da academia e dos cientistas, na condução de estudos bem delineados, que possam propor estratégias seguras, de orientação e prática de PRBE em pacientes pré e pós TOS, de grande abrangência.
