# Supplementary material for: Reabilitação Baseada em Exercícios para Pacientes Pré e Pós Transplante de Órgãos Sólidos
Source: Arq Bras Cardiol. 2022 Jul 29;119(2):255–6. [Article in Portuguese] doi: 10.36660/abc.20220373 (PMC9363049; doi:10.36660/abc.20220373)
Supplement: Translation

# Translation: Exercise-Based Rehabilitation for Pre- and Post-Solid Organ Transplant Patients

**Authors:** Rosane Maria Nery

**Affiliations:** 1 Porto Alegre RS Brazil Grupo de pesquisa em cardiologia do exercício (CardioEx), Hospital de Clínicas de Porto Alegre, Porto Alegre, RS – Brazil

**Keywords:** Organ Transplantation/rehabilitation, Transplantation Immunology, Physical Activity, Kidney Transplantation, Liver Transplantation

In the last decades, numerous advances in solid organ transplantation (SOT) have resulted in greater patient survival, resulting in a considerable increase in the number of transplants performed in the world.^1,2^ SOT is a life-saving intervention in patients with heart disease, pulmonary, renal or hepatic. Although the recipients improve both in functional capacity and quality of life (QoL), these are still not equivalent to the same levels of healthy individuals.^3^

The long waiting period, caused by the lack of organ donors, often makes patients unprepared for the transplant physically and mentally. In addition to the classic cardiovascular risk factors, we still have the patient’s lack of adherence to the programs, knowledge deficits about the rules of conduct after transplantation, non-acceptance of the new organ, fear of rejection, lack of a physical exercise routine, strategies for coping and occupational health issues and social rights.^4,5^

Rehabilitation is an essential part of contemporary care for patients before and after transplantation. The aim is to improve graft survival and reduce deaths from infection/rejection. Rehabilitation programs have prophylactic and therapeutic objectives, meeting the recommendations of maintaining improvements in QOL, reducing morbidity from cardiovascular diseases and improving long-term survival in transplant recipients.^6^ Therefore, greater attention should be given to postoperative interventions surgical procedures that help in the individualized management of these patients and that can result in a better prognosis.^7^

Physical training should be highlighted among non-pharmacological post-surgical interventions, as it is associated with significant improvement in exercise tolerance and functional capacity, reduced disability and decreased cardiovascular morbidity and mortality. This has also been shown to be beneficial in several groups of chronic diseases that can lead to SOT. It is known that there is a limitation in the ability to perform physical exercises in pre-SOT individuals, and most studies have focused on candidates for heart and lung transplantation.^8^ However, people with chronic kidney or liver disease also demonstrate limitations in pre-transplant exercise capacity, often due to secondary consequences of disuse, such as muscle weakness, rather than as a consequence of their primary disease process.^9^ In these individuals, peak oxygen consumption limitation seems to be related to peripheral muscle dysfunction and not to central factors, such as cardiovascular or respiratory limitations.^10^

Despite the evidence showing the potential benefits of physical exercise for both pre and post-SOT patients, there is a great lack of places that offer this care globally. This worsened after the COVID-19 event that further restricted access to rehabilitation centers. A large proportion of TOS recipients engage in low levels of physical exercise and face barriers to being physically active.

The study by Ribeiro et al.^11^ suggests a strategy where, after pre-participation assessment, in the absence of cardiovascular contraindication and according to the patient’s preference, he can choose to perform his exercise program at the hospital gym, at the gym community or at home. This model allows more people to engage in an exercise program, receive guidance from a qualified professional, and have periodic face-to-face or teleconsultation consultations.^12^ The results found by the authors reinforce the importance of the supervised program but emphasize that any type of treatment will be effective as long as the patient proposes to perform it.^11^ In addition to the limitations described by the authors, the fact that, in the same study, recipients of different organs were approached have a pathophysiological inheritance that can directly influence rehabilitation outcomes.

Larger, well-controlled studies of physical exercise that specifically include transplant candidates are still needed to propose specific guidelines on exercise dose and program duration to achieve the best benefits.^13^ Future studies on this topic should also focus on the effects of exercise during the waiting list period, preparing the patient for the transplant event and early post-transplant clinical outcomes.

For the world population, access to rehabilitation centers is still very limited, especially in places with a low structure and health resources and a low socioeconomic and cultural level. Our challenge is knowing how to use the knowledge of academia and scientists in conducting well-designed studies which can propose safe strategies, guidance and practice of PRBE in patients pre and post-SOT of great scope.
